# Case report: Suspected propofol associated Heinz body anemia in five mechanically ventilated dogs: a historical case series

**DOI:** 10.3389/fvets.2025.1500464

**Published:** 2025-02-03

**Authors:** Emily M. Ireland, Claire R. Sharp, Ellie M. Leister, Susan Boyd

**Affiliations:** ^1^Brisbane Veterinary Emergency and Critical Care Service, Albany Creek, QLD, Australia; ^2^School of Veterinary Medicine, Murdoch University, Murdoch, WA, Australia; ^3^Pet Intensive Care Unit, Underwood, QLD, Australia; ^4^Vetnostics Pathology, Murarrie, QLD, Australia

**Keywords:** oxidant, erythrocyte, eccentrocytosis, propofol, tick paralysis, snake envenomation, total intravenous anesthesia, transfusion

## Abstract

**Objective:**

The aim of this report is to raise awareness of the risk of oxidant-induced erythrocyte injury, including Heinz body (HB) anemia, in critically ill dogs by describing the condition in five dogs receiving constant rate infusions of propofol.

**Case summary:**

This case series describes five dogs with suspected propofol-induced HB anemia undergoing mechanical ventilation (MV) for lower motor neuron disease. Four of the five dogs were treated for tick paralysis (*Ixodes holocyclus*) and one was treated for suspected eastern brown snake (*Pseudonaja textilis*) envenomation. Propofol constant rate infusions were administered as part of total intravenous anesthesia. All five dogs became anemic, and a complete blood count and blood smear interpretation by a specialist clinical pathologist confirmed the presence of oxidative red blood cell injury (eccentrocytosis and HBs). The duration of MV ranged from 76 to 131 h, with HBs identified within 47–96 h of commencing propofol. All five dogs survived to discharge, with one dog requiring a blood transfusion.

**Discussion:**

While propofol-induced HB anemia is a recognized phenomenon in cats, to the author’s knowledge, this is the first case series detailing multiple occurrences in dogs. Veterinarians should be aware of the risk of propofol-induced oxidative erythrocyte injury in dogs receiving prolonged infusions of propofol, and consider risk mitigation by using propofol as part of multiagent intravenous anesthesia, keeping dose rates as low as possible, and daily monitoring of blood smears and red blood cell indices.

## 1 Introduction

Propofol (2,6-diisopropylphenol) hydrochloride is a non-barbiturate anesthetic agent commonly used in human and veterinary medicine. Favored for its short duration of action, smooth induction capabilities, and reliability as a titratable sedative, it is particularly useful in the critical care setting as a constant rate infusion (CRI) for dogs undergoing mechanical ventilation (MV) (1, 2).

Propofol undergoes hepatic, pulmonary, and renal metabolism, with renal excretion (3). Dual action GABA-*α* receptor agonist and NMDA antagonist produces reliable central nervous system depression with loss of consciousness at higher doses (2, 4). Although considered relatively safe and minimally cumulative with a short half-life and context-sensitive half-time, dose-dependent cardiovascular and respiratory depression may occur. This can be minimized with the implementation of multimodal total intravenous anesthesia (TIVA) with benzodiazepines, opioids, and/or dissociates for dose sparing effects (2, 5).

Propofol-associated Heinz body (HB) anemia is a specific adverse effect well noted in cats. Feline red blood cells (RBCs) are thought to be more sensitive to oxidative injury due to species-specific molecular differences in hemoglobin sulfhydryl groups and altered glucuronidation (4, 6–9). Apart from one published case of a dog receiving propofol and high volume intravenous lipid emulsion (IVLE) therapy for the treatment of 5-fluorouracil toxicosis (10), evidence of HB anemia and eccentrocytosis associated with propofol administration in dogs is lacking. HBs refer to a focal accumulation of oxidant-induced denatured hemoglobin that sticks to the erythrocyte cell membrane, giving the appearance of a small “blister” on the surface. These cells can then be removed from circulation by phagocytosis, thus resulting in anemia. Eccentrocytes are erythrocytes in which a part of the cell membrane adheres to itself, causing the concentrated hemoglobin molecules to shift to the remaining half of the cell (6, 7, 11).

This report describes the condition of five dogs with suspected propofol-associated HB anemia undergoing MV for lower motor neuron disease in an Australian intensive care facility. Samples were submitted to a clinical pathology laboratory^1^ for HB determination, with Wright and rhodanile blue stained smears prepared in a uniform manner and assessed by board-certified clinical pathologists.

## 2 Case report

### 2.1 Case 1

A 5-year 6-month-old female, spayed, cavalier King Charles spaniel (12.4 kg) was presented with 24 h of lethargy, inappetence, retching, and acute onset ataxia. Physical examination showed non-ambulatory tetraparesis, absent gag reflex, and moderately increased respiratory effort with dull lung sounds. An *Ixodes holocyclus* (IH) paralysis tick was removed, and tick antiserum^2^ (TAS) was administered (1 mL/kg IV once), along with the topical application of fluralaner acaricide^3^. Thoracic radiographs revealed right middle lobe alveolar infiltrates consistent with aspiration pneumonia and the dog was started on amoxicillin^4^ (22 mg/kg IV q 8 h). During the first 12-h of hospitalization, respiratory effort deteriorated and breathing became unsustainable, prompting endotracheal intubation and pressure-controlled ventilation (PCV+) MV. Initial settings included 100% FiO_2_, 5 cmH_2_O positive end-expiratory pressure (PEEP), 15 cmH_2_O inspiratory pressure (PInsp) above PEEP, and a respiratory rate (RR) of 30 bpm. Aggressiveness of ventilator settings were reduced as the dog stabilized with improved alveolar recruitment. FiO_2_ was weaned to 60% within 53 h, and 40% within 54 h of commencement of MV.

TIVA consisted of CRIs of propofol^5^ (0.05–0.1 mg/kg/min IV), midazolam^6^ (0.2–0.3 mg/kg/h IV), and butorphanol^7^ (0.2–0.3 mg/kg/h IV). Additional treatments included lactated Ringers’ solution^8^ (LRS) (1.9 mL/kg/h IV), potassium chloride supplementation^9^ (0.1–0.3 mmol/kg/h IV CRI), maropitant^10^_i_ (1 mg/kg IV q 24 h), and single doses of acepromazine^11^ (0.01 mg/kg IV), atropine^12^ (0.02 mg/kg IV), and furosemide^13^ (1 mg/kg IV). Serial electrolyte concentrations are summarized in Table 1. A bronchoalveolar lavage (BAL) was performed for culture and susceptibility (C&S) following development of leukopenia, pyrexia, and identification of septic suppurative respiratory secretions. Gram stain revealed large numbers of gram-positive cocci. Antibiotic therapy was escalated to include enrofloxacin^14^ (10 mg/kg IV q 24 h) and metronidazole^15^ (10 mg/kg IV q 12 h) pending C&S results. Bacterial culture grew *Staphylococcus pseudintermedius* that was pansusceptible, and *Enterococcus faecalis*, sensitive only to amoxicillin–clavulanic acid, chloramphenicol, and doxycycline.

**Table 1:** Summary statistics (Median, Min–Max) for electrolyte concentrations measured in five dogs receiving propofol infusion during mechanical ventilation, compared to the institutional reference interval.

Case number	Electrolyte concentration in mmol/L Displayed as median (Min–Max)				Count
	Sodium	Potassium	Chloride	Ionized calcium	
1	144 (139–147)	3.9 (2.7–5.2)	112 (103–116)	1.23 (1.15–1.3)	29
2	149 (144–159)	3.65 (2.8–4.7)	121 (114–134)	1.29 (1.16–1.36)	20
3	143 (141–149)	3.65 (3.2–4.3)	115 (111–120)	1.37 (1.23–1.45)	17
4	146.5 (142–150)	4.0 (3.2–5.1)	123 (114–127)	1.34 (1.28–1.43)	16
5	146 (137–149)	3.7 (2.3–4.4)	116 (110–121)	1.34 (1.09–1.55)	23
Reference interval	142–150	3.4–4.9	110–125	1.12–1.45	

**Figure 1:**
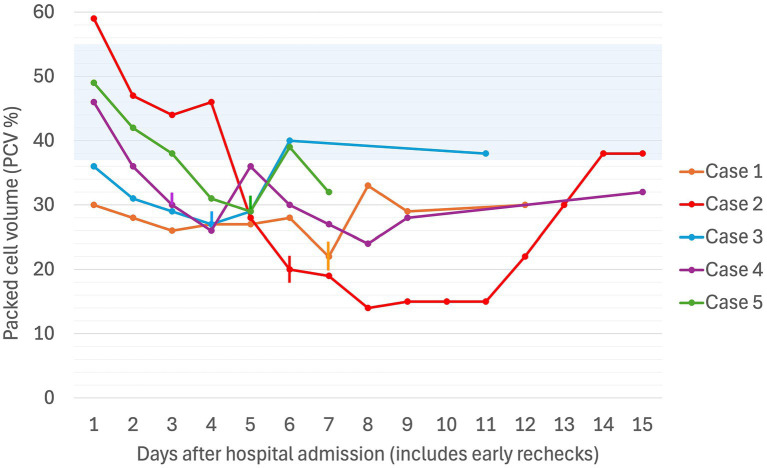
Serial packed cell volume (PCV in %) in five dogs experiencing Heinz body anemia associated with propofol infusion for mechanical ventilation. The blue shaded region is the reference interval for PCV (37–55%). Where multiple PCVs were recorded in a day, the highest value for the day was used. If a PCV was not recorded on a given day, the Hct was used. The vertical lines denote the day on which the propofol CRI was discontinued for each case.

**Figure 2:**
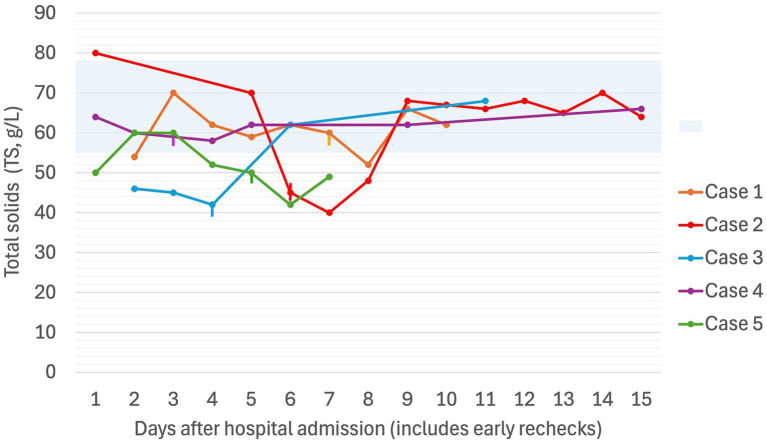
Serial total solids (g/L) in five dogs experiencing Heinz body anemia associated with propofol infusion for mechanical ventilation. The blue shaded region is the reference interval for TS (55–78 g/L). Where multiple TS were recorded in a day, the highest value for the day was used. The vertical lines denote the day on which the propofol CRI was discontinued for each case.

**Figure 3:**
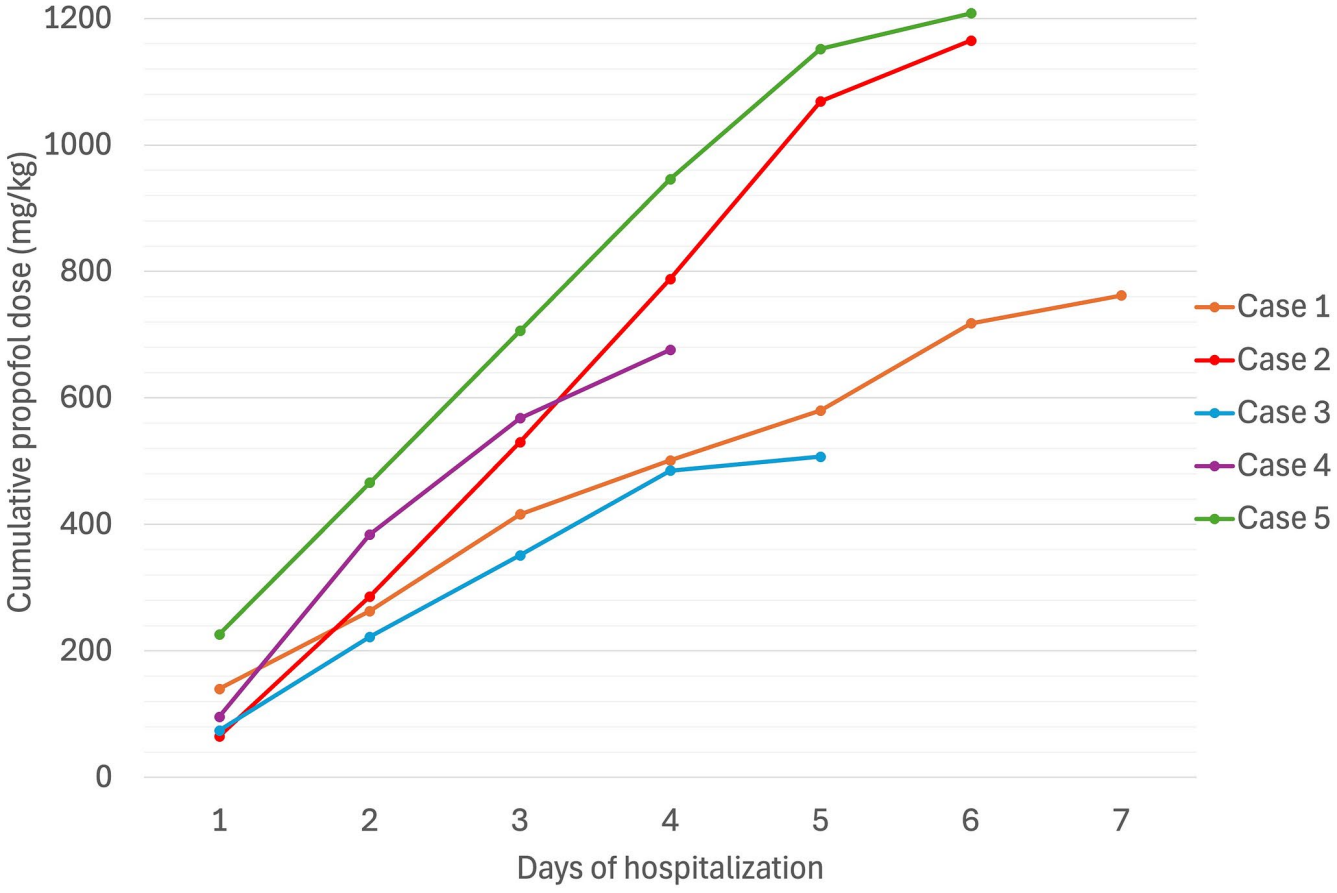
Cumulative propofol dose (mg/kg) in five dogs experiencing Heinz body anemia associated with propofol infusion for mechanical ventilation.

**Figure 4:**
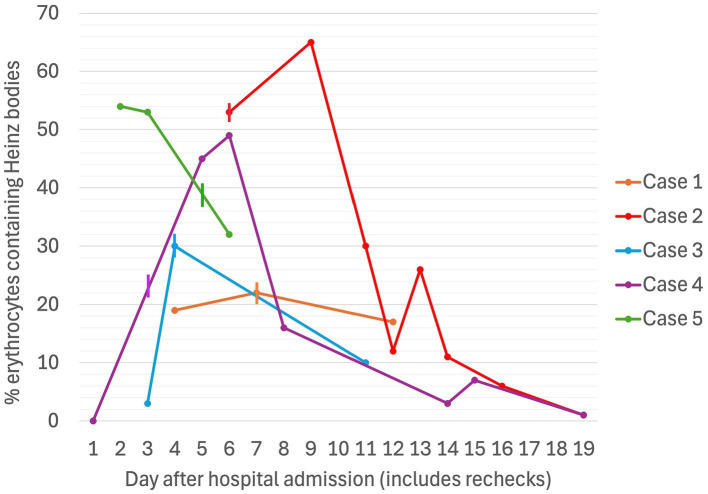
Serial percentages of erythrocytes containing Heinz bodies in five dogs associated with propofol infusion during mechanical ventilation. The vertical lines denote the day on which the propofol CRI was discontinued for each case.

**Figure 5:**
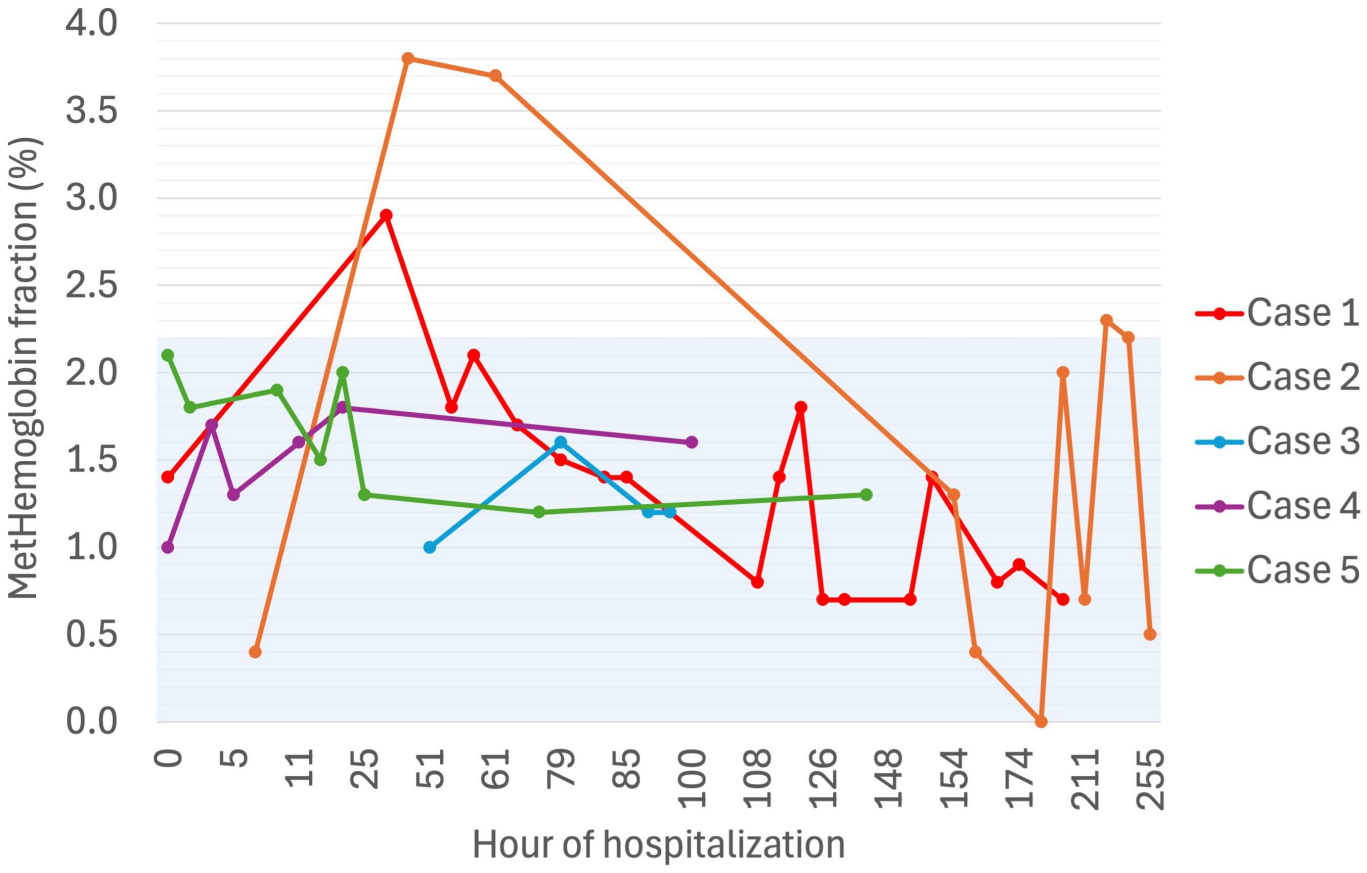
Serial methemoglobin fraction (%) in five dogs experiencing Heinz body anemia associated with propofol infusion during mechanical ventilation. The blue shaded region is the reference interval for methemoglobin fraction as published for 41 healthy dogs (25).

A non- or pre-regenerative anemia was documented on Day 1 (hematocrit [Hct] 30%, total solids [TS]/total protein [TP] not reported). Packed cell volume (PCV) decreased to 28% on Day 2 (TS 54 g/L), and further to a nadir of 22% on Day 7 (TS 52 g/L), before increasing back to 30% by Day 12 (serial PCV and TS as shown in Figures 1, 2). The recovery of anemia was spontaneous, and this dog did not require blood transfusion. HBs were first evident on Day 4 (identified in approximately 19% of erythrocytes), when the cumulative propofol dose was 444 mg/kg, increasing to 22% on the final day of MV (Day 7, cumulative dose 762 mg/kg, Figure 3), before decreasing to 17% on Day 12 (Figure 4). Methemoglobin (MetHb) fractions measured via blood gas analysis ranged from 0.7 to 2.9% over the duration of hospitalization (Figure 5). Mild anisocytosis and moderate eccentrocytosis were also documented on Day 4 and Day 7 as part of the complete blood counts, interpreted by a board-certified clinical pathologist, with only a small number of eccentrocytes remaining on Day 12. No spherocytes or ghost cells were visualized. Ambulation returned on Day 10 and the dog was discharged from hospital on Day 12. Upon telephonic follow-up, the owner reported a full recovery, however, the dog was lost to further laboratory assessment.

### 2.2 Case 2

An 8-year, one-month-old male, neutered, poodle (19.5 kg) was presented with 24 h of lethargy, progressive ataxia, and abnormal breathing. Physical examination revealed dehydration and marked proprioceptive deficits. An IH paralysis tick was removed, TAS administered (1 mL/kg IV once), and a tick clip performed prior to bathing in permethrin insecticidal solution^16^. Initial treatment included LRS (3 mL/kg/h IV), fentanyl^17^ (2 ug/kg/h IV CRI), metoclopramide^18^ (1 mg/kg/day IV CRI), and acepromazine (0.01 mg/kg IV once). Twelve hours after admission, the dog had an acute deterioration due to an aspiration event and was started on supplemental nasal oxygen. Twenty-four hours after presentation, the dog had progressed to non-ambulatory tetraparesis, regurgitated, aspirated, and developed respiratory failure necessitating MV (PCV+; PEEP 5–7 cmH_2_O; PInsp 11 cmH_2_O; RR 24–28 bpm; FiO_2_ 100%). Thoracic radiographs revealed a severe alveolar pattern in the right middle and cranial lung lobes consistent with aspiration pneumonia. Antimicrobial coverage was provided with ampicillin^19^ (22 mg/kg IV q8 h) and enrofloxacin (10 mg/kg IV q24 h). Bronchoalveolar lavage cytology revealed septic suppurative inflammation with low numbers of gram-positive cocci. Bacterial culture yielded multidrug-resistant coagulase-negative *Staphylococcus haemolyticus* that was susceptible only to amikacin, chloramphenicol, and doxycycline. FiO_2_ was weaned to 60% within 16 h, and 40% within 43 h.

**Figure 6:**
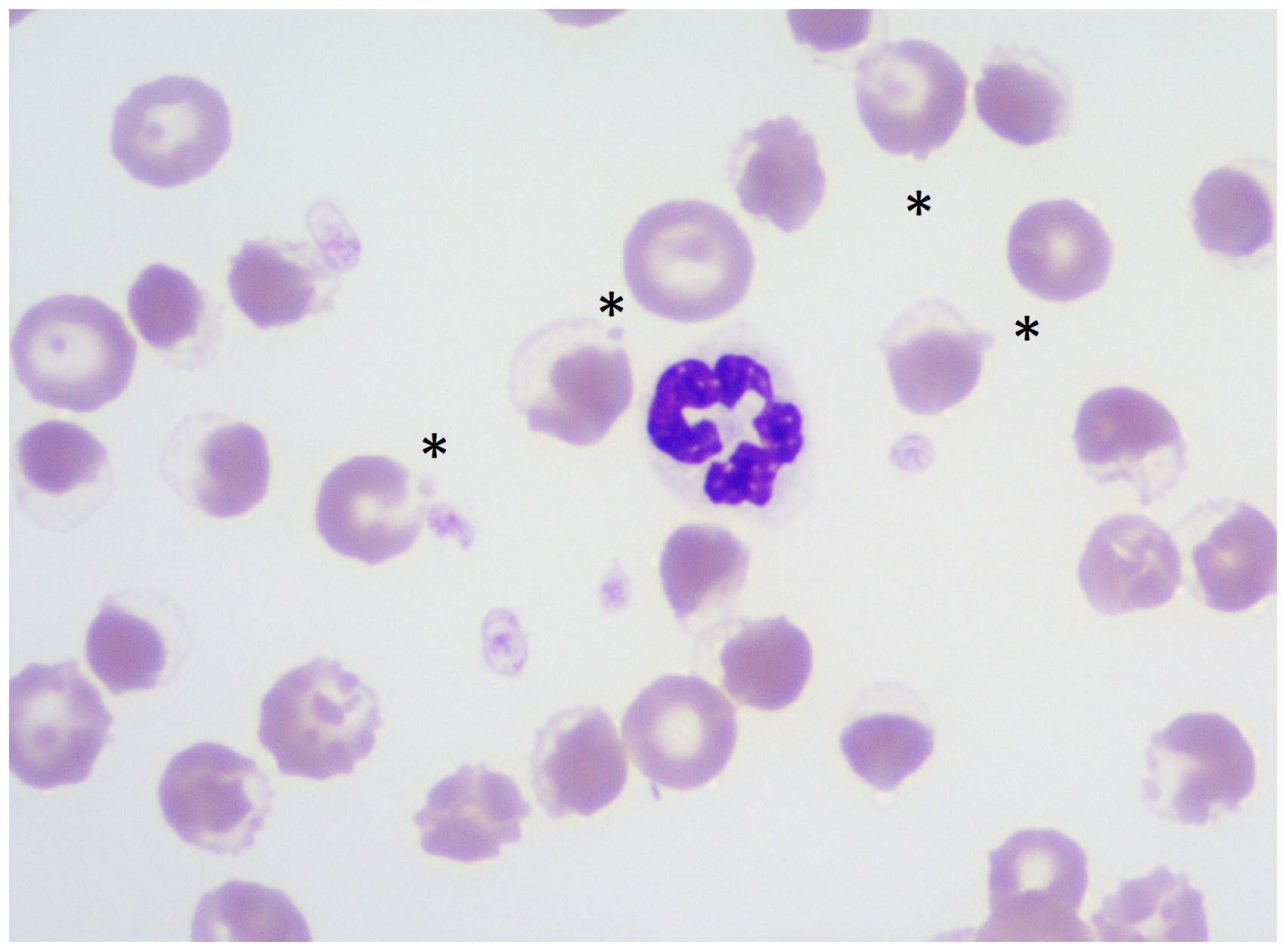
Wright stained blood smear demonstrating eccentrocytes and Heinz bodies (HBs) in a dog (Case 2) associated with propofol infusion. Magnification 1,000×. Asterisks show HBs projecting off eccentrocytes.

**Figure 7:**
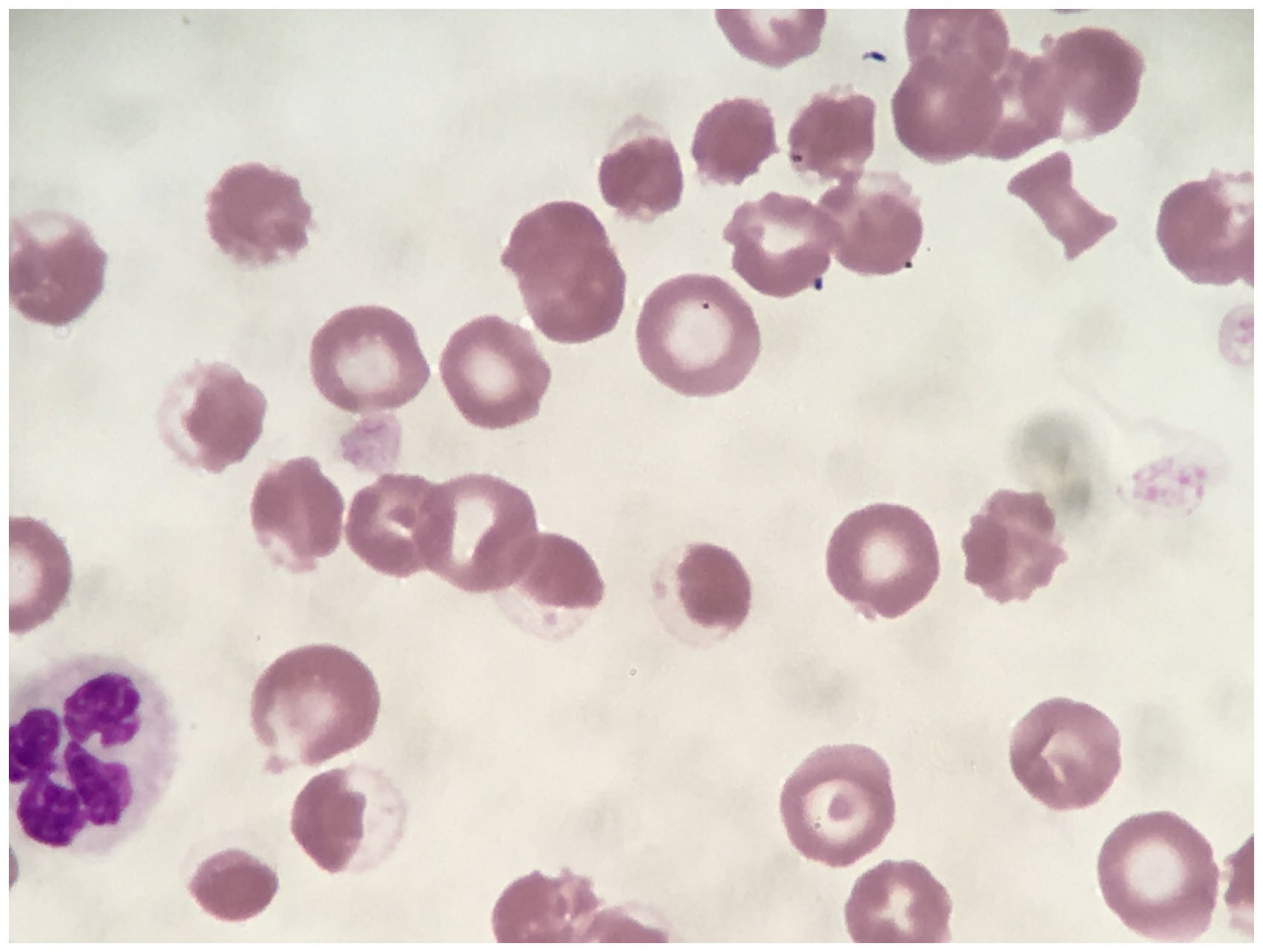
Rhodanile blue stained blood smear demonstrating eccentrocytes and Heinz bodies (HBs) in a dog (Case 2) associated with propofol infusion. Magnification 1,000×.

Initial TIVA consisted of propofol (0.15–0.2 mg/kg/min IV), midazolam (0.3 mg/kg/h IV), and fentanyl (3–8 ug/kg/h IV) CRIs, with butorphanol (0.2–0.3 mg/kg/h IV) replacing the latter on Day 2. PCV fell from 59% on Day 1 (TS 80 g/L) to 28% on Day 5 (TS 70 g/L, icterus), and further to 16% (TS 45 g/L) on Day 6, when hematology revealed a hematocrit of 13, 53% HBs, and marked eccentrocytosis (Figures 4, 6, 7). Due to concern for drug-induced oxidative cell injury, the propofol CRI was discontinued (cumulative dose 1,165 mg/kg) and replaced with alfaxalone^20^ (1–3 mg/kg/h IV CRI). Midazolam was replaced by medetomidine^21^ (1–3 ug/kg/h IV CRI) for deeper sedative effects and a nasogastric tube was placed for administration of gabapentin^22^ (10 mg/kg q8 h) and trazodone^23^ (5 mg/kg q8 h). This revised anesthetic protocol was continued until Day 7 when MV was discontinued. Ambulation returned on Day 12 of hospitalization. N-acetylcysteine^24^ (140 mg/kg IV once, followed by 70 mg/kg IV q6h for seven doses) was commenced.

Despite propofol discontinuation, hematology assessed by a specialist clinical pathologist on Days 7 and 8, documented a persistent severe anemia (Hct 14, 15%) with 65 and 30% HBs respectively, moderate eccentrocytosis and moderate anisocytosis. On Day 9, a single dose of human intravenous immunoglobulin (0.5 g/kg) was administered IV over 8 h. On Day 10, a single dose of dexamethasone (0.2 mg/kg IV) was also administered at the suggestion of the internal medicine service, albeit with the expectation that these were unlikely to have an effect on the anemia. Transfusion triggers were not evident until Day 11 of hospitalization (lowest PCV 12%, TS 65 g/L, icteric) at which time a packed RBC transfusion (8 mL/kg IV over 4 h) increased the PCV to 22% (TS 68 g/L). Thereafter, the PCV slowly increased spontaneously to 38% (TP 70 g/L) by hospital discharge on Day 14. MetHb fraction ranged from 0 to 3.8% over the duration of hospitalization (Figure 5). Serial electrolyte concentrations are summarized in Table 1. HB anemia further improved at the dog’s first recheck to hematology (Day 17, PCV 38%, TS 64 g/L, 6% HBs) and second recheck (Day 22, PCV 40%, TS 59 g/L, 1% HBs). At final recheck on Day 42, the canine patient was clinically normal, and there was complete resolution of oxidative changes (0% HBs), although the PCV was toward the lower end of the reference interval (39% [ref: 37–55%], TS 63 g/L).

### 2.3 Case 3

A one-year, 10-month-old male, entire Anatolian shepherd (75 kg) was presented with acute onset ataxia progressing to non-ambulatory tetraparesis. Tick prophylaxis was up-to-date, and an eastern brown snake (*Pseudonaja textilis*) was witnessed on the property 24 h before. Upon presentation, the dog was non-ambulatory and quickly developed unsustainable respiratory effort necessitating emergency intubation and MV (PCV+; PEEP 5 cmH_2_O; PInsp 11 cmH_2_O; RR 20–26 bpm; FiO_2_ 100%). Thoracic radiographs revealed right middle lung lobe alveolar infiltrates consistent with aspiration pneumonia and amoxicillin (22 mg/kg IV q8 h) was started. A presumptive diagnosis of eastern brown snake envenomation was made following a full body clip, negative tick searches, and the presence of a mild coagulopathy (activated clotting time 148 s [ref: 90–120]). Two vials of Tiger-Brown antivenom^25^ (8,000 IU tiger snake, and 8,000 IU brown snake antivenom IV), LRS (2–4.5 mL/kg/h), metoclopramide (1 mg/kg/day IV CRI), maropitant (1 mg/kg IV q24 h), and chlorpheniramine^26^ (0.5 mg/kg IM once), were administered. FiO_2_ was weaned to 60% by 12 h, and 40% by 23 h. A BAL for C&S was performed due to identification of persistent septic suppurative respiratory secretions, with amoxicillin replaced with piperacillin/tazobactam^27^ (80 mg/kg IV q8 h), which was continued following growth of susceptible *Pseudomonas aeruginosa*.

TIVA was maintained with CRIs of propofol (0.1 mg/kg/min IV), midazolam (0.2–0.4 mg/kg/h IV), fentanyl (3 ug/kg/h IV), and ketamine^28^ (0.3–0.5 mg/kg/h IV). Blood gas analysis performed during the first hour of MV reported a Hct of 36% with normal RBC morphology on smear, but Hct decreased to a nadir of 27% (TS 42 g/L) on Day 4. Blood samples collected after 52 h of propofol administration (cumulative dose 379 mg/kg) documented a mild, normocytic, normochromic anemia with 3% HBs, small numbers of Howell–Jolly bodies, and occasional eccentrocytes. Following client discussion, the decision was made to continue propofol since alternate anesthetic agents were not financially viable. The percentage of erythrocytes containing HBs increased to 30% on Day 4 (Figure 4). Urinalysis, thoracic and abdominal ultrasound did not identify evidence of hemorrhage, and normalization of clotting times (Prothrombin time [13.8; ref. 14–20 s], activated partial thromboplastin time [122.6; 94–123 s]) confirmed resolution of coagulopathy. MV was discontinued at hour-94 (cumulative dose 507 mg/kg). The dog’s anemia resolved without requirement for blood transfusion, with PCV increasing to 40% (TS 62 g/L) on Day 6. MetHb fraction ranged from 1.0 to 1.6% over the course of hospitalization (Figure 5). Serial electrolyte concentrations are summarized in Table 1.

Ambulation returned and the dog was discharged from hospital on Day 7. At a recheck on Day 11, physical examination was unremarkable and PCV somewhat stable at 38% (TS 68 g/L). Blood tests at this recheck documented a normochromic, regenerative anemia with mild anisocytosis, polychromasia, 10% HBs and small numbers of eccentrocytes. On account of his temperament, the dog was lost to further hematology follow-up; however, via regular telephone updates, the owner reported that he had made a full recovery.

### 2.4 Case 4

A 6-year-old male, entire Japanese Spitz (10 kg) was presented with 24 h of lethargy, vomiting, and generalized weakness progressing to tetraparesis and respiratory distress. Tick prevention was overdue. A tick clip located a tick crater, TAS was administered (1 mL/kg IV once), and topical fluralaner (see text footnote 3) was applied. A tick crater refers to the location where a tick embeds itself in the skin of a host, which develops a bowl shape with the tick in the center, and red, raised skin surrounding it. Palpebral and gag reflexes were absent. Generalized harsh bronchovesicular sounds with diffuse crackles were evident on lung auscultation, and pulse oximetry readings were 93–95% on FiO_2_ 21%.

Treatment included bilateral nasal oxygen supplementation (100 mL/kg/min), LRS (2.5 mL/kg/h IV), potassium chloride (0.1 mmol/kg/h IV CRI), amoxicillin (22 mg/kg IV q8 h), butorphanol (0.2 mg/kg/h IV CRI), maropitant (1 mg/kg IV q24 h), and metoclopramide (1 mg/kg/day IV CRI). Emergent intubation and MV was required following a regurgitation event on Day 1 (PCV+; PEEP 7 cmH_2_O; PInsp 11 cmH_2_O; RR 30 bpm; FiO_2_ 100%). The TIVA protocol included CRIs of propofol (0.13–0.16 mg/kg/min IV), butorphanol (0.3 mg/kg/h IV), and medetomidine (2–3 ug/kg/h IV). Right middle lung lobe aspiration pneumonia was evident on thoracic radiographs. Respiratory cytology identified septic suppurative inflammation, a BAL was performed, and enrofloxacin (10 mg/kg IV q24 h) commenced pending C&S. Bacterial culture yielded light mixed growth with no predominant organism isolated. FiO_2_ was weaned to 60% by 8 h, and 40% by 42 h.

PCV was 46% on Day 1 (TS 64 g/L), decreasing progressively to 25% on Day 3 when MV was discontinued (cumulative propofol dose 676 mg/kg). On Day 5, blood tests identified Hct 22% (TS 56 g/L), with moderate numbers of eccentrocytes and 45% HBs (Figure 4), described as punctate in size and often single or few per RBC. Further blood tests performed on Day 6 confirmed a mild anemia (Hct 26%, TP 52 g/L) with mild anisocytosis, 49% HBs, and numerous eccentrocytes. S-adenosylmethionine^29^ (SAMe) was started (22.5 mg/kg PO q24). Ambulation returned on Day 5 and the dog was discharged on Day 6. MetHb fraction ranged from 1.0 to 1.8% over the course of hospitalization (Figure 5). Serial electrolyte concentrations are summarized in Table 1. Hematology rechecks on days 8, 9, 15, 16 and 20 reported HB percentages of 62, 16, 3, 7, and 1%, respectively. Eccentrocytes were not present beyond Day 15 and Hct stabilized at 40% on Day 20. The dog made a full clinical recovery.

### 2.5 Case 5

A one-year, 9-month-old female, entire border collie (17 kg) was presented with vomiting and mild weakness following removal of an IH paralysis tick. On initial examination, the dog was weakly ambulatory with markedly increased respiratory effort. Treatment included TAS (1 mL/kg IV once), LRS (2 mL/kg/h IV), potassium chloride (0.1 mmol/kg/h IV CRI), butorphanol (0.1 mg/kg IV once), maropitant (1 mg/kg IV q24 h), metoclopramide (1 mg/kg/day IV CRI), and esomeprazole^30^ (1 mg/kg IV q12 h). Thoracic radiographs identified megaesophagus and severe aspiration pneumonia in the right middle, right cranial, and left cranial lung lobes, prompting commencement of antimicrobial coverage with amoxicillin (22 mg/kg IV q8 h), enrofloxacin (10 mg/kg IV q24 h), and metronidazole (10 mg/kg IV q12 h). Due to persistent hypoxemia, despite nasal oxygen therapy (100 mL/kg/min, bilaterally), PCV+ MV was initiated (PEEP 5 cmH_2_O; PInsp 10 cmH_2_O; RR 28 bpm; FiO_2_ 100%). Anesthesia was maintained with CRIs of propofol (0.14–0.17 mg/kg/min IV), midazolam (0.3 mg/kg/h IV), and butorphanol (0.2–0.3 mg/kg/h IV). FiO_2_ was weaned to 60% by 38 h and 40% by 93 h. A BAL was performed on Day 5, identifying septic suppurative inflammation with moderate numbers of gram-positive cocci. Antimicrobial coverage was escalated to meropenem^31^ (12 mg/kg IV q8 h) while awaiting results, which later identified multidrug-resistant, methicillin-resistant *Staphylococcus pseudintermedius,* and broadly susceptible *Escherichia coli*.

PCV decreased from 49% (TS 50 g/L) on Day 1 to 42% on Day 2 (TS 60 g/L), at which time after 47 h of MV (cumulative propofol dose 516 mg/kg) a pathologist report documented 54% HBs (Figure 4) and numerous eccentrocytes. PCV decreased further on Day 3 (38%, TS 60 g/L), with 53% HBs, reaching a nadir of 25% (TS 52 g/L) on Day 4. MV was discontinued at the end of Day 5 of hospitalization (cumulative propofol dose 1,208 mg/kg) and a final pathology assessment on Day 7 reported a normochromic, mildly regenerative anemia (Hct 32%, TS 49 g/L) with 32% HB and numerous eccentrocytes. MetHb fraction ranged from 1.2 to 2.1% over the course of hospitalization (Figure 5). Serial electrolyte concentrations are summarized in Table 1. The dog regained ambulation and was discharged from hospital on Day 8. Although no rechecks occurred, the owners reported the dog to have made a full recovery.

## 3 Discussion

This case series adds to the existing evidence from a single case report (10), that propofol can induce oxidant injury including HB hemolytic anemia and eccentrocytosis in dogs, suggesting that RBC oxidant injury is not isolated to cats. In most dogs in this case series, anemia was mild and self-limiting; however, one dog developed severe anemia requiring pRBC transfusion and protracted hospitalization.

Given the unusual nature of these cases, it was prudent to consider the pathophysiology of oxidative injury in general, and oxidative injury to erythrocytes specifically. Oxidative products including reactive oxygen species and reactive nitrogen species are produced in small quantities as a consequence of the life-sustaining process of cellular respiration, and in disease including pro-inflammatory states or systemic toxin exposure (12). Many protective antioxidant pathways exist to limit oxidative cellular damage. Erythrocytes have numerous protective enzymes such as reduced glutathione, superoxide dismutase, and methemoglobin reductase, which mitigate harmful oxidative effects from oxidative metabolites (7). Should these protective pathways become overwhelmed, oxidative cellular injury can result, manifesting with abnormal erythrocyte morphology most commonly in the form of eccentrocytes and HBs (6–8). Oxygen toxicity has been implicated as a potential cause of oxidative cellular injury (13, 14); however, direct RBC injury due to oxygen toxicity has not been described in critically ill humans or animal models of oxygen toxicity (15).

Three major mechanisms have been reported in the pathogenesis of oxidative erythrocyte damage, which can occur concurrently or in isolation. Firstly, direct cytotoxic cell membrane damage predominantly results in the formation of eccentrocytes with or without HBs. HB anemia has been previously reported in dogs secondary to toxicosis associated with zinc (16), skunk musk (17), onions (18), garlic and chives (19, 20), 5-hydroxytryptophan, and naphthalene (21), as well as treatment with the non-opioid analgesic metamizole/dipyrone (22) and exposure to fragrance products (23). Although the dogs in our study did receive many drugs as part of their treatment, none of the therapies they received have previously been reported to cause oxidative erythrocyte injury, and as such, propofol was suspected to be the cause. Additionally, oxidant injury characterized by eccentrocytosis has been documented associated with a variety of conditions in a large epidemiologic study in dogs (11). With the presence of both eccentrocytes and HBs in all dogs described in this case series, direct cytotoxicity is suspected.

Secondly, oxidative damage can occur within hemoglobin molecules with oxidation of ferrous iron to ferric iron, as seen in acetaminophen toxicity. This process predisposes to hemolysis and can result in a significant methemoglobinemia (6, 7), which was not identified to any great degree in our cases. One case report of chronic high dose acetaminophen administration in a dog (46 mg/kg PO q24 h for 6 weeks) demonstrated a metHb fraction of 6.4% (24). At the time of maximal HB populations, metHb fractions in our cases were 1.7, 0.7, 1.6, 2.0, and 1.3%, while HBs were present in 22, 51, 10, 65, and 53% of erythrocytes, respectively. While an institutional reference interval for metHb fraction has not been generated in the authors’ hospital, a previously published study has reported a reference interval of 0–2.2%, generated in 41 healthy dogs (25). In that same study, dogs with sepsis had metHb fractions up to 3.5% (25). The highest metHb fraction reached across all cases irrespective of HB populations was 3.8% (Case 2, Day 3 of MV). While the dog with the highest metHb fraction went on to develop the most severe anemia, he was not anemic at the time of this metHb fraction. Of the remaining four dogs in our case series, the next highest metHb fraction was 2.9%, which is similar to the higher metHb fractions reported in a large group of dogs hospitalized in an ICU setting (0.1–2.9%, *n* = 466) (26). The third mechanism of oxidative erythrocyte damage occurs on account of species differences in hemoglobin structure, with greater numbers of sulfhydryl groups potentiating oxidation. While canine hemoglobin contains two such groups, feline hemoglobin contains eight, rendering cats much more susceptible to oxidative damage than their canine counterparts. This has been implicated in acetaminophen toxicity and propofol-associated HB anemia in cats (4, 6–9, 27).

Additional mechanisms of oxidative erythrocyte injury have been identified in human studies with propofol shown to cause non-competitive inhibition of human erythrocyte glutathione, thereby reducing erythrocyte protective mechanisms against oxidative injury (28). Current literature suggests critical illness can result in reduced canine erythrocyte glutathione concentration in comparison to healthy dogs, with lower glutathione found to be associated with severity of illness and overall mortality (12). As an attempt to restore any potential glutathione deficiency leading to RBC membrane instability in the current study, two dogs were prescribed glutathione substrates (SAMe or N-acetylcysteine). By promoting glutathione production, both products have previously demonstrated beneficial antioxidant effects in the face of oxidative erythrocyte injury and glutathione depletion in dogs, cats, and humans (29–32). While N-acetylcysteine is the most recognized antidote for acetaminophen toxicity, SAMe has also been shown to normalize glutathione for this purpose (30). Nonetheless, a small clinical study failed to identify an improvement in redox status or clinical outcome in systemically ill, hospitalized dogs treated with antioxidant supplementation (33). Given the retrospective observational nature of this report, it is impossible to determine if administration of a glutathione precursor changed the clinical course of the dogs in our case series that received it. Future directions from this case series could include prospective randomized clinical trials investigating the potential for antioxidants to mitigate oxidative erythrocyte injury in dogs receiving propofol infusions for MV in an ICU setting.

In contrast to dogs, low numbers of HBs are not uncommon in healthy cats as the unique non-sinusoidal structure of the feline spleen impairs their ability to remove abnormal erythrocytes from circulation (6, 7). Greater numbers of sulfhydryl groups within feline hemoglobin molecules also potentiate RBC oxidation in comparison to dogs and humans (6, 7). HBs have been reported in numerous conditions in cats including diabetes mellitus, lymphoma, and hyperthyroidism, whereas eccentrocytes are more commonly reported in dogs (11). In addition, the feline liver is not able to metabolize numerous drugs and toxins through the normal pathway of glucuronidation, resulting in a greater reliance on alternative pathways (oxidation and sulfation)—an important feature applying to acetaminophen and propofol (8).

In cats, repeated or prolonged propofol infusions are contraindicated due to their predisposition to developing HB anemia (9, 27). A study investigating the effects of repeated propofol anesthesia in six healthy cats receiving 6 mg/kg induction doses, followed by 30-min CRIs (0.20–0.30 mg/kg/min) for up to seven consecutive days identified a significant increase in HBs (27). HBs increased after Day 3 (cumulative propofol dose 36–45 mg/kg), reaching a mean of 31% HBs across all cats by Day 7 (cumulative dose 84–105 mg/kg). Clinical signs of illness including malaise, anorexia, diarrhea, and facial edema resolved 24–48 h after discontinuation of propofol administration, but resolution of HBs was not monitored (27). A more recent case report documented a cat undergoing 20-fraction radiation for management of fibrosarcoma that developed significant HB anemia following 12 consecutive propofol anesthetics (9). After a cumulative propofol dose of 62.4 mg/kg, the PCV fell from 38 to 22% with up to 50% of RBCs containing HBs. As with the aforementioned study, clinical signs of lethargy and anorexia resolved within 1 week of propofol discontinuation, at which point the PCV had increased to 30% and HB were no longer present (9). The authors of this cat case report suggested that the total accumulated dose as well as the duration of propofol administration perhaps contributed to propofol-induced HB formation (9), but studies exploring serial propofol administration in cats undergoing radiation therapy have failed to identify propofol-associated hematologic changes, when compared to other anesthetic protocols that did not use propofol (34, 35). Nonetheless, consistent with the greater sensitivity of cats to propofol, the dogs in our study had received much higher cumulative propofol doses before HBs were first identified.

No dogs in this case series exceeded the maximum rate of propofol infusion (0.8 mg/kg/min) recommended for procedural anesthesia (36), with actual dosages administered ranging from 0.05 to 0.2 mg/kg/min. However, specific maximum doses of propofol infusion for the purposes of MV, that may occur for days to weeks, have not been published. In human medicine, due to the concern for propofol infusion syndrome, some authors recommend against propofol infusion for more than 48 h or rates >0.067 mg/kg/min (4 mg/kg/h) (37, 38).

Pharmacokinetic studies of propofol in dogs have demonstrated a mean elimination half-life of 322.3 ± 22.7 min after a 4 mg/kg IV induction dose, followed by 0.4 mg/kg/min CRI for approximately 1 h in healthy beagle dogs (39). Despite this study being conducted in a homogenous dog population with a standardized propofol dose (in 6/7 dogs), there was marked inter-individual variation in propofol blood concentrations. Interestingly, despite rapid anesthetic recovery, propofol was measurable in the blood of all dogs for 25 h, suggesting that propofol may accumulate in tissues during infusion (39). Given the varied signalment and heterogenous nature of the clinical disease in the five dogs reported in our case series, even more marked inter-individual variation is likely to have occurred. A long elimination half-life may explain why the proportion of HBs increased in some dogs after cessation of propofol infusion. Another possible explanation for this observation is simply that the peak proportion of HBs was missed due to the infrequent hematology.

Studies in humans also demonstrate a long elimination half-life of propofol (40, 41). A study of 7 males receiving propofol infusion rates of 0.058–1 mg/kg/min (3.5–6.2 mg/kg/h) for a median of 4.3 h (Min–Max 2.5–9.1 h) had a median elimination half-life of 26.6 h (Min–Max 13.1–44.7 h) (40). The authors describe that blood concentrations of propofol fell rapidly in the initial period after cessation of the propofol infusion, but thereafter the rate of decline was slower such that propofol was still measurable 95 h later (40). Another pharmacokinetic study in humans investigated three infusion rates of propofol; 3 mg/kg/h (0.05 mg/kg/min), 6 mg/kg/h (0.1 mg/kg/min), and 9 mg/kg/h (0.15 mg/kg/min), with 6 patients per group receiving an infusion of at least 2 h duration (41). This study reported that the propofol pharmacokinetics were best explained by a three-compartment open model with a long terminal elimination phase (terminal half-life 355 ± 227 min), that was not statistically, significantly different among the three dose groups (41). The authors of both studies describe that the high lipid solubility of propofol results in an extremely large volume of distribution and that the ultimate slow elimination is explained by slow return of the drug from the tissues (40, 41). A large prospective study is required in dogs to assess propofol pharmacokinetics associated with prolonged infusions, and to better establish whether a relationship exists between propofol CRI rate, cumulative dose, or duration of exposure in dogs, and erythrocyte oxidative injury.

Existing literature on the occurrence of erythrocyte oxidative injury in dogs is sparse. Caldin et al. performed a large-scale retrospective study analyzing clinical and laboratory data from 4,251 dogs to describe the occurrence and severity of eccentrocytosis (11). Eccentrocytes were identified in 60 (1.4%) dogs, 40 (66.6%) of which had mild-to-moderate anemia (11). A statistically greater proportion of dogs with diabetic ketoacidosis, T-cell lymphoma, and vitamin K antagonism had eccentrocytes, compared to dogs without these diseases. Other drugs or toxins associated with the presence of eccentrocytes included propofol (*n* = 5), non-steroidal anti-inflammatory drugs (5), azathioprine (1), cyclosporine (1), onion (3), and garlic (1) (11). Unlike our study, specialized stains were not routinely used to assess for the presence of HB, therefore while eccentrocytes were identified as the predominant oxidative change in diseases usually associated with HB formation in cats, the authors acknowledged that the presence of HB may have been understated. Oxidative changes were reported to resolve within 24–72 h following diagnosis and specific treatment (11), in contrast to our study where resolution of eccentrocytes and HBs was more protracted.

The cases described in this case series have some similarities and some differences to the previously published single case report of a dog with oxidative RBC damage associated with the administration of propofol and IVLE as part of treatment for 5-FU toxicosis (10). While dogs in both reports received propofol CRIs for prolonged periods, the dose, duration, and indications were different between studies. The case reported by Romans et al. received a higher propofol rate (0.2 mg/kg/min, up to 0.36 mg/kg/min) but for a shorter duration (40 h) (10), than our cases (0.05–0.2 mg/kg/min, for 76–131 h). Although the total propofol exposure was not reported by Romans et al., it was likely lower than that in our cases. The indication for propofol was also different (control of status epilepticus in Romans et al. vs. anesthesia for MV in our case series). Nonetheless, dogs in both reports were considered critically ill, a factor potentially contributing to the risk of oxidant injury (12).

The case reported by Romans and colleagues received additional lipid in the form of IVLE (2 doses of 21.6 mL/kg), which the authors state is higher than the recommended doses in humans (10). While IVLE is reported to cause hemolysis in dogs*, ex vivo* this effect occurs within 1 h (42), as opposed to the delayed onset in the case report (4 days after the discontinuation of propofol infusion). The case reported by Romans et al. also had evidence of erythrocyte injury at presentation, in the form of echinocytosis, suspected to be due to 5-FU toxicosis, which was not evident in our cases. That being said, one dog in our case series (Case 1) had a mild anemia of unknown origin at baseline, despite being clinically well prior to the onset of tick paralysis. Another possible risk factor for oxidant injury in the case by Romans et al. was that of severe persistent electrolyte abnormalities including hyponatremia (minimum reported value 123 mmol/L), hypokalemia (minimum 2.5 mmol/L), and ionized hypocalcemia (minimum 0.66 mmol/L) (10). While the dogs reported herein had some electrolyte concentrations outside of the reference interval, abnormalities were not as severe or persistent as reported by Romans et al., and thus are not expected to have contributed to oxidant-induced erythrocyte injury.

Our cases had variation in the proportion of erythrocytes containing HBs, with a maximum of 65% in Case 2, compared to 22% in Case 1. In Cases 1, 2, 3 and 5, HB anemia was first detected while the dogs were still receiving propofol infusions; however, in Cases 2 and 3, the proportion of RBCs containing HBs increased after discontinuing propofol. Case 4 was more similar to the case reported by Romans et al., where HBs were not detected until after discontinuation of propofol infusion. We were fortunate in our cases to have hematology assessed by a clinical pathologist for more objective quantification of the severity of HBs, as compared to the semi-quantitative 2–3/hpf reported by Romans et al. (10). Like the case reported by Romans et al., all of the dogs that we reported also had eccentrocytosis, but in contrast, none of our cases had spherocytosis. It is possible that the cells referred to as spherocytes in Romans et al. were pyknocytes, i.e., remnants of eccentrocytes after losing their extruded membrane flaps, since the remaining smaller cells with condensed HB can resemble spherocytes. While oxidative RBC injury was identified in our cases, the extent to which it contributed to anemia is difficult to determine since critically ill dogs often develop a degree of anemia through multiple mechanisms. Potential mechanisms contributing to anemia in the dogs reported in this case series may have included anemia of inflammatory disease, blood loss from serial blood collection, erythrocyte sequestration in the spleen or non-splenic sites secondary to anesthesia, and/or anesthesia-related vasodilation and fluid redistribution (43, 44).

HB hemolytic anemia has also recently been described in a series of 13 hospitalized dogs receiving metamizole, a non-opioid analgesic with antipyretic and spasmolytic effects (22). In this study, HB formation was noted within 3–10 days from the commencement of metamizole, with 28–95% HB reported and no correlation identified between daily dose administered, HB populations, or severity of anemia. An immediate decrease in HBs was noted following discontinuation of the medication. Some of these findings align with our case series, where HBs were first identified between 2 and 6 days of propofol commencement and no apparent correlation could be made between dosage, extent of oxidative cell injury, severity of anemia, and requirement for blood transfusion therapy. Similarly, oxidative changes were shown to reduce in all patients following discontinuation of propofol; however, a notable lag period was appreciable in some cases prior to resolution.

Our investigation of these cases also led us to the human literature on adverse effects of propofol. A rare complication in humans receiving prolonged administration of propofol during MV is referred to as propofol-related infusion syndrome. Propofol infusion syndrome is associated with multiple organ dysfunction, with specific components including cardiovascular dysfunction, dyslipidemia, acid–base and electrolyte disturbances (particularly acidosis and hyperkalemia), rhabdomyolysis, and acute kidney injury (37, 38, 45). The occurrence of this condition has also been suggested in a dog (46), but it does not seem to be associated with HB anemia and is not consistent with the adverse effects experienced by the dogs in this case series.

The clinicopathologic findings of the cases reported herein are most consistent with primary oxidative erythrocyte injury; however, other causes of anemia, such as hemolysis secondary to erythrocytic parasites were not specifically ruled out since tick-borne disease is considered rare in this area. Technically it is possible that dogs in this case series may have had tick-borne hemotropic parasites that went undetected, since *Babesia vogeli* (but not *B. gibsoni,* or *B. canis*), *Anaplasma platys* (but not *A. phagocytophilum*), *Mycoplasma haemocanis*, and various candidatus hemotropic mycoplasmas (e.g., *C. mycoplasma haematoparvum*, and C. *Mycoplasma haemobos*) have been detected very occasionally by PCR in dogs in this region (south-eastern Queensland) (47). Nonetheless, the nature of anemia in the dogs described in this case report lacked evidence of immune-mediated erythrocyte destruction (e.g., spherocytes, positive saline agglutination test) classically seen with *Babesia* spp. and *Mycoplasma haemocanis* spp. infection, and the presence of HBs are not seen secondary to hemotropic parasites. Other factors that make it less likely that erythrocytic parasites were the cause of anemia include that no parasites were seen on the blood smear cytology evaluated by specialist clinical pathologists at any time. Anemia was not present at the time of hospital admission, except in Case 1, and anemia improved in all cases following cessation of propofol infusion, despite no specific treatment for hemotropic parasites.

Fortunately, despite the development of anemia, the dogs in our case series all survived to hospital discharge. This is somewhat consistent with the generally good prognosis for dogs with tick paralysis and snake envenomation undergoing MV (48–50). The development of aspiration pneumonia is also commonly reported in dogs with tick paralysis, and the spectrum of organisms cultured in our cases are similar to those reported previously (49).

Limitations of our report are related to its nature as a historical case series. Blood tests, including full hematology, were performed at attending clinicians’ discretion. Since routine daily testing was not performed, delayed detection of oxidative changes may have occurred, and peak population of HBs may have been missed. Additionally, the time to resolution of HBs could also not be quantified for most cases as limited hematological follow-up was available following hospital discharge. Another limitation is that this study was not designed to determine the incidence of oxidant-induced erythrocyte injury in dogs receiving propofol. It is possible that the phenomenon is much more common, and potentially milder, as we did not screen all dogs receiving propofol infusions during MV. Future studies should investigate the incidence of oxidant-induced erythrocyte injury (HBs, eccentrocytes), its relationship to anemia, and prospectively assess biomarkers of oxidative stress in dogs receiving propofol infusions in an ICU setting. Biomarkers of oxidative stress that could be investigated, and that have been investigated in other studies in dogs, include measurement of reactive oxygen species in erythrocytes, reduced glutathione concentrations, vitamin E concentrations, glutathione peroxidase activity, total antioxidant capacity, and urinary 15-F_2_-isoprostanes (51, 52). Studies of propofol pharmacokinetics after prolonged intravenous infusion are also warranted in dogs.

Considering the findings in this report, clinicians should be vigilant in monitoring for development of oxidative erythrocyte injury, characterized by HB anemia and eccentrocytes, in dogs receiving prolonged infusions of propofol in the critical care setting. Daily assessments of PCV/TP and blood smears should be encouraged to assess for markers of oxidative injury, with routine CBC interpretations by specialist pathologists highly recommended. While all five dogs in this report survived to discharge, 1/5 (20%) required a blood transfusion, and further cases have since been identified at the authors’ institution. As oxidative erythrocyte injury has the potential to increase morbidity and contribute to mortality in ICU patients, it is prudent for clinicians to minimize propofol infusion doses, by using multimodal TIVA. A larger cohort study is required to better ascertain the incidence and mechanism behind these oxidative changes and to develop strategies to minimize the risk this poses to our canine patients.

## Data Availability

The raw data supporting the conclusions of this article will be made available by the authors without undue reservation.

## Footnotes

^1^QML Pathology Vetnostics, 11 Riverview Place, Metroplex on Gateway, Murarrie, QLD, 4172.

^2^AVSL® Ixodes Holocyclus Antivenom; Australian Veterinary Serum Laboratories, Lismore, QLD, AUS.

^3^Fluralaner (Bravecto®); MSD Animal Health. Bendigo East, VIC, AUS.

^4^Amoxicillin (as Sodium) (Ibiamox®), USP 1g for injection; Juno Pharmaceuticals Pty Ltd, Cremome, VIC, AUS.

^5^Propofol (Propofol-Lipuro 1%®), USP 10mg/mL; B. Braun Australia Pty Ltd. Bella Vista, NSW, AUS.

^6^Midazolam (Midazolam Alphapharm®), USP 50mg in 10mL; Mylan Australia. Carole Park, QLD, AUS.

^7^Butorphanol (ilium Butorgesic Injection®), USP 10mg/mL; Troy Laboratories Pty Ltd, Glendenning, NSW, AUS.

^8^Lactated Ringers Solution (Hartmann’s®); Baxter Healthcare Pty Ltd. Toongabbie, NSW, AUS.

^9^Potassium Chloride, USP 10mmol (0.75g) in 10mL; Pfizer Australia Pty Ltd. Sydney, NSW, AUS.

^10^Maropitant Citrate (Cerenia®), USP 10mg/mL; Zoetis Australia Pty Ltd. Rhodes, NSW, AUS.

^11^Acepromazine (A.C.P 2®), USP 2mg/mL; Ceva Animal Health Pty Ltd. Glenorie, NSW, AUS.

^12^Atropine (ilium Atrosite®), USP 0.65mg/mL; Troy Laboratories Pty Ltd, Glendenning, NSW, AUS.

^13^Frusemide (ilium Frusemide Injection®), USP 50mg/mL; Troy Laboratories Pty Ltd, Glendenning, NSW, AUS.

^14^Enrofloxacin (Enro®) USP 50mg/mL; Headway Animal Health, Pemulwuy, NSW, AUS.

^15^Metronidazole, USP 5mg/mL; Baxter Healthcare Pty Ltd. Toongabbie, NSW, AUS.

^16^Permethrin (Permoxin®), USP 40g/L; Dermcare-Vet Pty Ltd, Slacks Creek, QLD, AUS.

^17^Fentanyl (Fentanyl Citrate®) USP 500ug in 10mL; Panpharma GmbH, Trittau, Germany.

^18^Metoclopramide (Metomide®) USP 5mg/mL; Ceva Animal Health Pty Ltd, Glenorie, NSW, AUS.

^19^Ampicillin (as sodium) (Austrapen®), USP 1000mg for injection; Mylan Australia. Carole Park, QLD, AUS.

^20^Alfaxalone (Alfaxan®), USP 10mg/mL; Jurox Pty Ltd. Rutherford, NSW, AUS.

^21^Medetomidine (ilium Medetomidine®) USP 1mg/mL; Troy Laboratories Pty Ltd, Glendenning, NSW, AUS.

^22^Gabapentin (APO-Gabapentin®), 100mg capsule; Apotex Pty Ltd. Macquarie Park, NSW, AUS.

^23^Trazodone hydrochloride, USP 100mg compounded tablet; Bova Australia. Caringbah, NSW, AUS.

^24^Acetylcysteine (Acetylcysteine-Link®), USP 2g in 10mL; Link Medical Products Pty Ltd. Warriewood, NSW, AUS.

^25^Tiger-Brown Snake Antivenom 8000 units®; Padula Serums Pty Ltd. Bairnsdale, VIC, AUS.

^26^Chlorpheniramine (ilium Histamil®), USP 10mg/mL; Troy Laboratories Pty Ltd, Glendenning, NSW, AUS.

^27^Piperacillin Sodium/Tazobactam Sodium (PipTaz-AFT®), USP 2.25g; AFT-Pharmaceuticals Pty Ltd, North Ryde, NSW, AUS.

^28^Ketamine (as Hydrochloride), USP 100mg/mL; Randlab Australia Pty Ltd, Revesby, NSW, AUS.

^29^S-Adenosylmethionine & Silybin (Denamarin®); Nutramax Laboratories Veterinary Sciences, INC. Lancaster, SC, USA.

^30^Esomeprazole sodium (Nexium®) USP 40mg powder for injection; AstraZeneca. Macquarie Park, NSW, AUS.

^31^Meropenem (as trihydrate) (Meropenem GH®), USP 500 mg powder for injection; Generic Health Pty Ltd. Box Hill, VIC, AUS.
